# The uses and abuses of the coherence – correspondence distinction

**DOI:** 10.3389/fpsyg.2015.00507

**Published:** 2015-04-30

**Authors:** Andrea Polonioli

**Affiliations:** Department of Philosophy, University of EdinburghEdinburgh, UK

**Keywords:** coherence, correspondence, bias, adaptive behavior, rationality, goals

## Abstract

Kenneth Hammond introduced a distinction between coherence and correspondence criteria of rationality as a tool in the study of judgment and decision-making. This distinction has been widely used in the field. Yet, as this paper seeks to show, the relevant notions of coherence and correspondence have been progressively considered to be too narrow and have undergone non-trivial conceptual changes since their original introduction. I try to show, first, that the proliferation of conceptualizations of coherence and correspondence has created confusion in the literature and that appealing to such notions has not helped to elucidate discussions over the nature of rational judgment and decision-making. Nevertheless, I also argue for a reframing of the debate. In fact, what seems to underlie several contemporary appeals to the notions of coherence and correspondence is best explained in terms of a contrast between what I call rule-based and goal-based rationality. Whilst these categories do need further refinement, they do seem to be useful for organizing and understanding research on rational judgment and decision-making.

## Hammond’s Coherence – Correspondence Distinction

In recent decades, research on judgment and decision-making has witnessed the development of several different approaches to human rationality, which differ in terms of the importance they attribute to traditional normative models and the adaptiveness of behavior in the assessment of performance (e.g., Kahneman and Tversky, 1972; Gigerenzer et al., 1999; Chater and Oaksford, 2000). Whilst these different projects have arguably remained somewhat disconnected, Hammond (1990, 1996) has attempted to remedy this balkanization. To promote cross-fertilization between different lines of research, he first tried to outline a framework that could allow him to identify different strategies available to us in the study of human judgment. He introduced a distinction between coherence and correspondence criteria of rationality, where the latter strategy is called correspondence “because it evaluates the correspondence between the judgment and the empirical fact that is the object of the judgment” (Hammond, 2007, p. 16). Coherence, on the other hand, relates to the fit between people’s judgments. Specifically, Hammond (2007, p. 16) defines coherence as “the consistency of the elements of the person’s judgment.” According to him, “it is easy to see the difference between a judgment that is directed toward coherence – make it all fit together – and one that is directed toward the correspondence between a judgment and a fact” (Hammond, 2007, p. 19).

Armed with this distinction, Hammond tried to make sense of different lines of research in the study of human judgment. On one extreme, Brunswik’s (1956) research offered a prominent illustration of work on correspondence. His research focused entirely on empirical accuracy, which comes down to the correspondence between a judgment and an object. On the other extreme, Hammond argued that research in the heuristics and biases tradition provided a paradigmatic example of research on coherence (Gilovich et al., 2002). Whilst the heuristics and biases project was motivated by a desire to offer accurate descriptions of human judgment and decision-making and give insight into underlying mechanisms and processes, its researchers measured behavior against a set of normative principles. For example, subjects are said to violate coherence when they commit the conjunction fallacy, ranking the conjunctive event A and B as higher in probability than one of its component events (see, e.g., Tversky and Kahneman, 1983). Hammond concluded that this distinction proved useful in categorizing research in the field. Yet, he was aware that, while correspondence refers here to empirical accuracy, a criterion with which we are all familiar, providing a specific characterization of coherence would be a daunting task.

## A Reformed Distinction

Hammond’s coherence – correspondence distinction has been widely recognized as a useful conceptual tool in the study of judgment and decision-making (Adam and Reyna, 2005; Mandel, 2005; Newell, 2005; Baron, 2012; Lee and Zhang, 2012; Wallin, 2013). It was also celebrated in 2009 in a special issue of the journal *Judgment and Decision Making*. What I want to stress, here, is that Hammond’s original distinction has not been just adopted, but also significantly adapted in the literature, as the original concepts of coherence and correspondence have seemed to be too narrow.

For instance, consider that the distinction has attracted particular attention from Gigerenzer, Hertwig, and other cognitive psychologists and evolutionarily behavioral scientists at the Centre for *Adaptive Behavior and Cognition* and at the *Centre for Adaptive Rationality*. On Hammond’s view, these scholars exemplify research focusing on correspondence. In this context, Hammond’s distinction was not used to promote cross-fertilization between different research projects. In fact, these theorists used the distinction to interpret the philosophical import of their research on fast-and-frugal heuristics and to question the normative commitments typically shared in the field. Previous research in the sciences of decision-making had shown that people often avoid making trade-offs among attributes (Payne et al., 1993; Gowda and Fox, 2002; Kahneman and Frederick, 2002). As Gigerenzer (2000, p. 191) put it, compensation is often seen to be “the cornerstone of classical rationality, assuming that all commodities can be compared and everything has its price.” While these patterns of behavior seemed irrational to researchers engaged in bias research, heuristics that violate principles such as compensatoriness and transitivity have been shown to outperform inferences that do not imply such violations. Overall, these heuristics seem fast, frugal, and yet accurate (for some criticisms see, e.g., Bröder, 2000; Newell et al., 2003), and the resulting behavior has been described as ecologically rational, viz. adapted to the environment in which humans act (Rieskamp and Reimer, 2007, p. 273).

Notably, such findings have been interpreted by appealing to Hammond’s distinction and as suggesting that greater coherence does not always imply greater correspondence, and *vice versa* (Katsikopoulos, 2009). Moreover, these theorists stress that “the function of heuristics is not to be coherent” (Gigerenzer et al., 1999, p. 22), and that it is correspondence that counts for the assessment of the ecological rationality of people’s behavior.

It appears clear that coherence has been used in a quite broad sense here, and that the concept has become a rather complex and heterogeneous mixture, encompassing different constituents such as compensatoriness and transitivity. To some extent, it should come as no surprise that this notion has been reshaped. After all, Hammond’s original definition of coherence as the internal fit of someone’s judgments was admittedly vague and thus allowed for quite flexible use. Furthermore, if Hammond’s intention in introducing the concept of coherence was to characterize the normative perspective adopted by projects such as that of heuristics and biases, his focus on how judgments fit together looks rather narrow, for instance because those theorists were also interested in assessing decisions, and not only judgments. In addition, Hammond’s concept of coherence might be of little help when trying to apply it to different and more complex tasks. While these may be good reasons to find Hammond’s original concept of coherence to be too narrow, we must note that these broader characterizations of coherence do not bear much resemblance to Hammond’s original concept. While transitivity does have to do with coherence in the usual sense of the word, compensatoriness does not. In fact, some would say that non-compensatory strategies could be perfectly coherent as a matter of logical consistency and of coherence of preferences.

Some scholars have explicitly attempted to redefine the notion of coherence as coherence between judgments and some rational principles, moving beyond Hammond’s previous characterization in terms of a fit between people’s judgments. For instance, Newell (2005, p. 11), in an attempt to explicate Hammond’s original concept, writes that “human judgment can be evaluated by the degree to which it coheres with a formal model.” Along similar lines, Stevens (2008, p. 291) points out that “human judgment can be evaluated by the degree to which it coheres with a formal model, such as Bayes theorem.” What is evident is that the concept of coherence has undergone a quite radical conceptual change.

Changes have been even more radical with regard to the concept of correspondence. Some authors have preserved Hammond’s characterization. For instance, Stevens (2008, p. 291) claims quite clearly that “correspondence refers to the degree to which decisions achieve empirical accuracy; that is, whether they reflect the true state of the world.” But others have used the concept of correspondence more broadly, stressing that “there are multiple correspondence criteria relating to real-world decision performance” (Todd and Gigerenzer, 2000, p. 738), where these encompass empirical accuracy, speed, frugality, fitness-maximization, and successful exchanges with others.

These semantic changes do not have to be seen as an oversight or as resulting from a lack of care in the application of Hammond’s original concept. Researchers attempting to amend Hammond’s concept of correspondence have found the original characterization to be too narrow. On the one hand, Hammond (2007, pp. 78–79) identified empirical accuracy as the hallmark of adaptive behavior. For instance, he stressed that “the theory of evolution rests on natural selection, and natural selection—apparently—rests on *fitness,* that is, good, accurate empirical correctness.” Also Brunswik (1956), thought by Hammond to offer paradigmatic research into correspondence, seemed to highlight a strong link between adaptive psychological processes, and their being conducive to empirical accurate judgments. But the links between empirical accuracy and adaptiveness are controversial. While Gigerenzer et al. (1999) stress the adaptive value of empirical accuracy, they also emphasize the importance of other goods. In particular, inspired by Simon’s work on bounded rationality, and focusing on the cost of searching for information when dealing with quite complex tasks, these scholars also stress the adaptive importance of saving time and energy. This suggests that links between accuracy and adaptiveness can be problematized, and research on fast-and-frugal heuristics suggests that it might not be adaptive to pay attention to empirical accuracy only. Importantly, however, while Gigerenzer et al. (1999) claim that the fast-and-frugal heuristics we use entail very small losses in accuracy, other evidence does not sit well with the claim that empirical accuracy is conducive to adaptive behavior. Convincing cases have been made for the claim that empirical inaccuracy might be evolutionarily adaptive (Haselton et al., 2009; McKay and Dennett, 2009). But empirical inaccuracy may not only be evolutionarily adaptive; it might also promote prudential goals (Taylor, 1989) or epistemic goals (Vallinder and Olsson, 2014).

As we have seen, there may have been good reasons for taking Hammond’s original concept of correspondence to be too narrow and for some researchers attempting to extend its scope. However, it is important to stress that these semantic changes have led to confusion. It is true that conceptual change may be part of scientific progress, but the problem here is that it is now unclear what researchers really mean when they use the notion of correspondence. Some scholars follow Hammond’s original distinction, while others do not.

It is also unclear to which sorts of behavior and phenomena correspondence might apply. Hammond and other scholars take the notion of correspondence to apply solely to beliefs. As Stevens (2008, p. 291) pointed out, “both criteria (coherence and correspondence) apply to inferences; preferences, however, have no correspondence criteria.” The underlying argument is the following. You might ask, for instance, whether someone’s preferences are transitive or not. But preferences cannot be assessed in terms of empirical accuracy. In brief, only beliefs can be true of the real world; desires and preferences cannot. Interestingly, however, if there are multiple and different correspondence criteria, it is unclear why correspondence should apply solely to beliefs. The idea that standards of accuracy might be applied to preferences and desires as well is not new. Dennett (1987, p. 49), for example, stressed that “a system’s desires are those it ought to have, given its biological needs and the most practical means of satisfying them.” But while it is true that empirical accuracy does not apply preferences, other criteria of correspondence could apply to preferences as well. This comes out quite clearly when we consider the criterion of maximizing fitness. Take, as an illustration, the case of mate preferences. Evolutionary psychologists have investigated these at length (e.g., Buss, 1989; Kenrick and Keefe, 1992). Mate preferences can affect the current direction of sexual selection by influencing who is differently excluded; they may also reflect prior selection pressures, and exert selective pressures on other components. Preferences can vary in their degree of adaptiveness.

## Rule-Based and Goal-Based Rationality

We now find ourselves in a quandary. On the one hand, attempts to reshape the notions of coherence and correspondence have led to confusion in the literature. However, there seem to be sensible reasons to think that Hammond’s original concepts are too narrow. How to remedy this predicament? In what follows, I argue that a reframing of the debate is advisable.

Hammond’s original concepts of coherence and correspondence have proven to be limited tools for organizing research on judgment and decision-making, and scholars should not seek to broaden such notions, but instead refrain from using them. In fact, what underlies several current appeals to the coherence – correspondence distinction seems to be best explained in terms of a contrast between what I will dub rule-based and goal-based rationality.

To support my conceptualization of this distinction, recall, first, that coherence is currently often interpreted as coherence with some sets of rational norms. Moreover, correspondence is often taken to encompass several goods and goals beyond empirical accuracy. This seems to point to a contrast between two different approaches to the assessment of rational behavior and cognition. One based on conformity with rational norms, typically associated with consistency and the axioms of subjective probability theory and of rational choice, and one based on the achievement of relevant goals and desirable outcomes. In fact, some theorists have explicitly associated correspondence with the achievement of a goal and stressed that relevant goals “go beyond accuracy, frugality, and making fast decisions” and “include transparency, group loyalty, and accountability” (Gigerenzer and Gaissmaier, 2011, p. 471). Moreover, other scholars appealing to the coherence – correspondence distinction have presented it as a “distinction between the assessment of a process and the assessment of an outcome” (Lee and Zhang, 2012, p. 366), where the contrast between assessing an outcome and assessing a process resembles the contrast between rule-based and goal-based rationality that I have just introduced.

This contrast is not exhaustive. For instance, one could assess behavior by focusing on intellectual virtues such as open-mindedness and attentiveness. However, a contrast between rule-based and goal-based rationality seems to underlie other binary distinctions made in the literature to highlight differences between normative approaches. For instance, Evans and Over (1996, p. 8) famously distinguished between personal rationality (rationality 1) and impersonal rationality (rationality 2). They characterize the former as thinking or “acting in a way that is generally efficient for achieving ones goals,” and the latter as “thinking or acting when sanctioned by a normative theory.” Moreover, Samuels et al. (2004) appealed instead to a distinction between deontological and consequentialist approaches to rational behavior. According to the deontologist, what it is to reason correctly is to reason in accord with some appropriate set of rules or principles, while another prominent view, often referred to as consequentialism, maintains that what it is to reason correctly is to reason in such a way that you are likely to attain certain goals or outcomes. While these distinctions might present differences, they revolve around a contrast between two different questions: is the organism following the relevant norm of rationality? Is the organism achieving its goal?

I want to suggest here that debates over the nature of rational judgment and decision-making can benefit from being reframed using the suggested distinction between rule-based and goal-based rationality. Consider that, since the introduction of Hammond’s original distinction, the number of different research programs concerned with rational judgment and decision has grown rather than decreased. In this babel, *ecological rationality* (Gigerenzer et al., 1999), *grounded rationality* (Elqayam, 2012), *rational analysis* (Chater and Oaksford, 2000), and *quantum cognition* (Pothos and Busemeyer, 2013) have emerged as some of the leading projects. Psychological research has traditionally assessed both judgements and decisions against the so-called “standard picture” (Stein, 1996, p. 4) of rationality, according to which behavior should be assessed against rules of first order logic, probability theory, and expected utility theory (cf. Baron, 2004). Others have questioned whether we should take as benchmarks of rationality classical principles of probability or whether quantum probability is a viable alternative theory (Pothos and Busemeyer, 2014).

However, other scholars concerned with the adaptiveness of people’s behavior have questioned the very idea that behavior and cognition should be assessed against some rational rules, and emphasized instead that behavior has to be ultimately assessed against goals. For instance, as we have already seen, Gigerenzer et al. (1999, p. 364) stress that “ecological rationality depends on decision- making that furthers an organisms adaptive goals.” In this spirit, some researchers have argued that violations of standard norms of rationality can lead not only to grater empirical accuracy, speed and frugality – as already suggested by research on fast-and-frugal heuristics – but also to other goals, such as, for instance, accountability (Lenton et al., 2013). But also advocates of other projects have stressed the importance of goal attainment: according to (Elqayam and Evans, 2011, pp. 236–237) “behavior typically well adapted and conducive to people’s personal goals can be described as rational.” An appeal to the importance of goals has characterized the project of *rational analysis* too: Chater and Oaksford (2000, p. 93) present their empirical program as “attempting to explain why the cognitive system is adaptive, with respect to its goals”, although they suggest that people’s success at achieving goals is due to their following standard rational principles. I suggest that scholars engaged in the “rationality debate” focus on my suggested distinction between rule-based and goal-based rationality: this distinction helps to classify and organize research on judgment and decision-making and is a more useful conceptual framework than Hammond’s original one, since key disagreements seem to rest upon whether behavior has to be assessed against a set of rational norms or against an organism’s relevant goals, and whether following standard principles of rationality leads to the achievements of goals. Moreover, while the contrast between rule-based and goal-based rationality underlies other distinctions offered in the literature, such as that between personal and impersonal rationality, the labels of rule-based and goal-based rationality seem to better capture the nature of the bones of contention between different projects in this literature that have been mentioned above.

At the same time, my suggested distinction needs further refinement. For instance, caution is required when considering the perspective of goal-based rationality. According to this view, to ask whether an organism’s behavior is rational means to ask whether the organism’s goal is being achieved. However, the notion of goal in contemporary psychology is not as clear as one might want (cf. Castelfranchi, 2014, p. 105), and different research projects may conceptualize it differently. This is not hair-splitting. For instance, the notion of rationality is often applied to non-human animals as well (Stanovich, 2013; Stevens and King, 2013), but it would then be important to have a conceptualization of what it means to achieve a goal or follow a rule that could be applied to non-human animals too. Importantly, different answers with regard to these issues would point to quite different perspectives on human rationality. By identifying these open questions to address, it is then possible to prompt theorists engaged in these debates to state their commitments more clearly and to set the agenda for future research.

## Conflict of Interest Statement

The author declares that the research was conducted in the absence of any commercial or financial relationships that could be construed as a potential conflict of interest.
